# Distinctive Feature of Microbial Communities and Bacterial Functional Profiles in *Tricholoma matsutake* Dominant Soil

**DOI:** 10.1371/journal.pone.0168573

**Published:** 2016-12-15

**Authors:** Seung-Yoon Oh, Jonathan J. Fong, Myung Soo Park, Young Woon Lim

**Affiliations:** 1 School of Biological Sciences, Seoul National University, Seoul, Republic of Korea; 2 Science Unit, Lingnan University, Tuen Mun, New Territories, Hong Kong; Woosuk University, REPUBLIC OF KOREA

## Abstract

*Tricholoma matsutake*, the pine mushroom, is a valuable forest product with high economic value in Asia, and plays an important ecological role as an ectomycorrhizal fungus. Around the host tree, *T*. *matsutake* hyphae generate a distinctive soil aggregating environment called a fairy ring, where fruiting bodies form. Because *T*. *matsutake* hyphae dominate the soil near the fairy ring, this species has the potential to influence the microbial community. To explore the influence of *T*. *matsutake* on the microbial communities, we compared the microbial community and predicted bacterial function between two different soil types—*T*. *matsutake* dominant and *T*. *matsutake* minor. DNA sequence analyses showed that fungal and bacterial diversity were lower in the *T*. *matsutake* dominant soil compared to *T*. *matsutake* minor soil. Some microbial taxa were significantly more common in the *T*. *matsutake* dominant soil across geographic locations, many of which were previously identified as mycophillic or mycorrhiza helper bacteria. Between the two soil types, the predicted bacterial functional profiles (using PICRUSt) had significantly distinct KEGG modules. Modules for amino acid uptake, carbohydrate metabolism, and the type III secretion system were higher in the *T*. *matsutake* dominant soil than in the *T*. *matsutake* minor soil. Overall, similar microbial diversity, community structure, and bacterial functional profiles of the *T*. *matsutake* dominant soil across geographic locations suggest that *T*. *matsutake* may generate a dominance effect.

## Introduction

The pine mushroom, *Tricholoma matsutake* (S. Ito and S. Imai) Singer, is an important ectomycorrhizal fungus in Asia due to its value as a food product [1, 2]. Fruiting bodies of *T*. *matsutake* grow in a fairy ring (also called a shiro) where hyphae aggregate densely with soil in the shape of ring around ectomycorrhizal host trees (Pinaceae and Fagaceae) [1, 3]. In hopes of successful cultivation, previous research focused on abiotic factors such as climate [4–6] and nutritional condition [7–9] for understanding the ecology of *T*. *matsutake*. However, studies on the biotic interaction of *T*. *matsutake*, especially microbial interaction, are limited.

Microbial interaction may be limited due to the dominance effect of *T*. *matsutake* in the fairy ring. Ohara and Hamada [10], using a culture-dependent method, found no bacteria in the fairy ring. Subsequent studies using culture-dependent [11], traditional molecular [12, 13], and next generation sequencing (NGS) based methods [14] corroborated the results of Ohara and Hamada [10], finding lower microbial diversity or abundance within the fairy ring of *T*. *matsutake*. In contrast, Kim et al. [15] found no difference in bacterial diversity between the fairy ring and non-fairy ring soil, with both having high diversity.

Together with reduced diversity, the dominance of *T*. *matsutake* may shape a distinct microbial community. Although previous studies found specific microbes present in the fairy ring of *T*. *matsutake* [11–13, 16], some studies used culture-dependent methods, which may have underestimated microbe diversity. Culture-independent and high-throughput technology (e.g. NGS) is a useful tool to fully understand microbial characteristics in *T*. *matsutake* dominant soil. Preliminary studies of microbial communities in the fairy ring demonstrated the usefulness of NGS technology [14, 15], and the next step is to extend analyses to include multiple samples across geographic locations to verify trends.

Although NGS has revealed high microbial diversity in ecosystems [17], functional analyses are needed to understand the role of microbes in the ecosystem. While metagenomics, metatranscriptomics, and functional arrays are good approaches for functional analysis [18, 19], these methods are constrained by cost and difficulty [20, 21]. Recently, Langille et al. [22] developed the software Phylogenetic Investigation of Communities by Reconstruction of Unobserved States (PICRUSt), which analyzes microbial function by combining phylogenetic analyses and genome database data to predict functional profiles. To date, PICRUSt has contributed to our understanding of microbial functional profiles in the animal gut [23], human mouth [24], ocean [25], and soil [26].

Our main objective was to explore the microbial characteristics associated with *T*. *matsutake* by comparing the diversity and community structure of bacteria and fungi, as well as bacterial functional profiles in *T*. *matsutake* dominant (*Tm*-dominant) soil and minor (*Tm*-minor) soil near the fairy ring. A dominance effect of *T*. *matsutake* would be implied if microbial characteristics were similar across multiple *Tm*-dominant soils, but different from the *Tm*-minor soil. We used pyrosequencing because it has proven to be a powerful method for revealing microbial interactions [27, 28], and expanded on the design of past studies by comparing multiple soil types from two geographic locations. Additionally, we used PICRUSt to predict and compare bacterial functional profiles of soil bacteria in the *Tm*-dominant soil. We expect this study to contribute to our understanding of the ecology of *T*. *matsutake* and microbial interactions in the natural environment.

## Materials and Methods

### Sampling location and strategy

In 2011, sampling was conducted during the fall, when *T*. *matsutake* produced fruiting bodies. Samples were collected from two locations in South Korea that are separated by ~220 km: Gyeongju (35° 48' 46"N, 129° 13' 48"E) and Hongcheon (37° 41' 35"N, 127° 58' 51"E). Sampling sites in Gyeongju and Hongcheon are research forests of government institutes (Gyeongsanbuk-do Forest and Environment Research Institute, and National Institute of Forest Science, respectively). Approval for sampling was granted by each institute. Both locations consisted of mixed forest dominated by the Japanese red pine (*Pinus densiflora*), oak trees (*Quercus* spp.), and ericaceous shrubs (*Rhododendron* spp.). For collecting the *Tm*-dominant soil samples, we conducted sampling near the fairy ring. Generally, abundance of *T*. *matsutake* is high in the fairy ring with whitish-gray colored soil and hyphae aggregations. We took three soil samples at 30 cm intervals near the fairy ring, and conducted sampling twice for each geographical location. In total, 12 soil samples taken from Hongcheon and Gyeongju were transported on ice and stored at -4°C for a maximum of one week before DNA extraction.

It has been shown that morphological characterization of the fairy ring may not correspond to soil dominated by *T*. *matsutake* if multiple fungal species co-exist [29]. Therefore, we confirmed soil type based on the sequence read abundance. We categorized soil samples into two types based on the proportion of *T*. *matsutake* sequences in the NGS analysis: *Tm*-dominant soil (≥50%) and *Tm*-minor soil (<50%). We added a status code (*Tm*-dominant soil: D, *Tm*-minor soil: m) to end of the sample name to denote its classification after sequence based confirmation (S1 and S2 Tables).

### DNA extraction, PCR amplification, and Pyrosequencing

For each sample, genomic DNA was extracted from 500 mg of soil using the FastDNA^TM^ SPIN kit for soil (MP Biomedicals, USA) following the manufacturer’s instructions. For fungi, the nuclear ribosomal internal transcribed spacer (ITS) region was amplified using ITS1F and ITS4 primers [30], while for bacteria, the 16S ribosomal DNA (16S) region was amplified using 27F [31] and 518R primers [32]. Amplicon libraries were prepared using primers with a 454 pyrosequencing adaptor and multiple identifier (MID) tag. The PCR program was as follows: 5 min at 94°C; 35 cycles of 30 s at 94°C, 30 s at 55°C, and 40 s at 72°C; and a final extension step of 5 min at 72°C. PCR products were confirmed using gel electrophoresis and purified using the HighPure^TM^ PCR Product Purification Kit (Roche, Germany). Individual PCR products were quantified using a NanoDrop spectrophotometer (Thermo, USA) and separately pooled for bacteria and fungi. Pyrosequencing was performed with a 454 GS Junior platform (Roche, USA) at ChunLab (Seoul, Korea). DNA was sequenced only in the reverse direction, and data were deposited in the NCBI Sequence Read Archive (SRP046049).

### Bioinformatics process and statistical analysis

Raw data were processed with QIIME 1.8.0 [33]. Reads having sequences shorter than 200 bp, an average nucleotide quality score under 25, ambiguous nucleotides, or mismatches in the MID tag were excluded from analyses. The remaining reads had MID tags removed and were denoised using Denoiser v1.7.0 [34] implemented in QIIME. Different workflows were used for the fungal and bacterial dataset; the bacterial workflow was chosen so that we could perform a functional prediction analysis. For the fungal dataset, chimeric sequences were removed and fungal operational taxonomic units (fOTUs) were clustered with 97% similarity using the USEARCH v5.2.236 [35] implemented in QIIME. Universal singleton fOTUs in total of samples were discarded from analyses. Taxonomy was assigned to fOTUs using the RDP classifier [36] to query the representative sequence (the most abundant sequence) against the UNITE database v. 7.0 [37]. For the bacterial dataset, we clustered bacterial OTUs (bOTUs) by using the open reference OTU picking process in UCLUST [35] with 97% similarity based on the Greengenes database (version May 2013) [38]. The final identification of bOTUs was assigned using the RDP classifier with an 80% confidence threshold based on the EzTaxon database [39]. The representative sequences of bOTUs were aligned to the Greengenes core alignment dataset using PyNAST [40]. Chimeric sequences were detected and removed using ChimeraSlayer [41]. Columns of data with only gaps were filtered from the alignment and a phylogeny was constructed using FastTree [42]. As for fOTU, universal singleton bOTUs were removed from analysis.

Before further analysis, we normalized sequences using the minimum number of sequences. Alpha diversity indices (Chao1, Shannon, and Shannon’s equitability) and taxonomic composition were calculated in QIIME. Before performing calculations for fungi, we removed sequences of *T*. *matsutake* to eliminate the estimation bias from the *T*. *matsutake* abundance. In addition to the other diversity indices, phylogenetic diversity index (PD) [43] was calculated for bacteria. Statistical analyses (ANOVA and Wilcoxon rank sum test) and graphical plots (boxplots and principal coordinates analysis [PCoA]) were performed using R v. 3.1.0 [44]. We used ANOVA for comparing diversity indices of microbial communities and a Wilcoxon rank sum test for comparing the OTU richness at the phylum level. PCoA plots were drawn using the phyloseq package with Bray-Curtis distances for fungi and weighted UniFrac distances for bacteria [45]. We tested the influence of two factors: soil type (the *Tm*-dominant soil and *Tm*-minor soil) and geography (Hongcheon and Gyeongju). To determine the explanatory coverage of the factors, we performed Constrained Analysis of Principal coordinates (CAP) analyses for CAP models specified in two ways: both factors together (~soil type + geography) and constrained to only one factor. In models constrained to one factor, we eliminated the effect of the other factor by assigning a conditioned factor (~geography+ Condition[soil type] and ~soil type + Condition[geography]). To test for the statistical significance of each CAP model, we performed an ANOVA with 999 permutations. We tested for statistical difference of microbial communities between samples using PERMANOVA (adonis) with 999 permutations implemented in QIIME, and compared the distribution of genera between soil types using a discovery odds ratio test in the metagenomeSeq package [46]. P-values in the discovery odds ratio test were corrected using the false discovery rate (FDR) of Benjamini and Hochberg [47]. To avoid misinterpretation, we considered only significant results for common and major genera (present more than 1% in at least three samples of one of the soil types) that had sufficiently large changes (|log_2_ Odds ratio| >1).

Functional profiles of bacterial communities were predicted using PICRUSt [22]. Since fungal genome data are currently limited, we performed PICRUSt analyses only for bacterial communities. We followed the tutorial instructions on the PICRUSt website for our workflow. Briefly, we predicted gene family profiles and 16S copy number by constructing a phylogeny with representative bOTU and Greengenes sequences for which genome data were available, followed by an ancestral state reconstruction. Metagenome prediction of bacterial communities was conducted using the calculated dataset after normalizing for 16S copy number. We obtained Nearest Sequenced Taxon Index (NSTI) scores for evaluating the accuracy of predicted metagenome profiles [22], with a low NSTI indicating high accuracy. The functions of predicted metagenomes were categorized with the KEGG pathways database [48]. We compared bacterial functional profiles at KEGG modules levels 4. We used the edgeR package [49] for analyzing differential abundance of modules by soil type. After normalizing module abundance using the trimmed mean of M values (TMM) method in edgeR, we retained modules with more than 100 counts per million (CPM) in the 3 samples at least, and measured fold changes of modules between the *Tm*-dominant soil and *Tm*-minor soil. Lastly, an exact test was conducted with multiple test correction using the FDR of Benjamini and Hochberg. We retained statistically significant modules with large fold change (|log_2_ fold change| >4).

## Results

A total of 237,107 fungal and 111,374 bacterial sequence reads were obtained, and after filtering 76,294 and 40,558 reads were retained respectively (S1 and S2 Tables). The proportions of *T*. *matsutake* reads in each sample were calculated for classification of soil types. In Hongcheon, *T*. *matsutake* reads were dominant (>80%) in H2 and H5, and almost nonexistent (0–1 reads) in the remaining four samples (H1, H3, H4, H6). In Gyeongju, three samples (G1, G2, and G3) were dominated by *T*. *matsutake* reads (>80%), but G4 and G6 (<2%), and G5 (34.7%) had a low proportion of *T*. *matsutake*. Therefore, we confirmed the samples to be five *Tm*-dominant soil (G1D, G2D, G3D, H2D, H5D) and seven *Tm*-minor soil samples (G4m, G5m, G6m, H1m, H3m, H4m, H6m) (S1 and S2 Tables).

### Microbial diversity associated with *T*. *matsutake*

#### Fungal diversity

**Fig 1:**
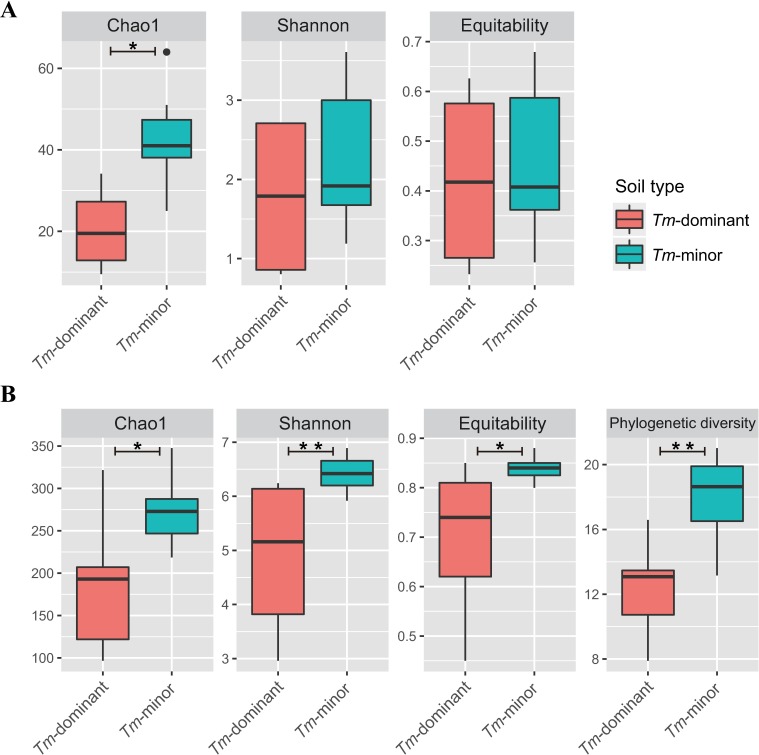
**Diversity indices of *Tm*-dominant and *Tm*-minor soil samples for (A) fungi and (B) bacteria.** All calculations were performed on normalized data. (*: *Corrected P* < 0.05, **: *Corrected P* < 0.01).

**Fig 2:**
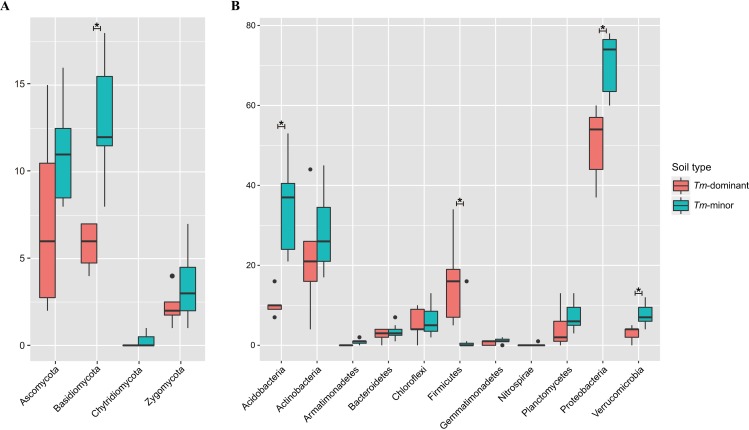
**OTU richness at the phylum level in *Tm*-dominant and *Tm*-minor soil samples of (A) fungal and (B) bacterial communities.** Significance of differential richness between the *Tm*-dominant and *Tm*-minor soil was evaluated using Wilcoxon rank sum test. The phyla that were significantly different between the soil types (*Corrected P* < 0.05) are represented with an asterisk.

A total of 76,294 reads and 187 fOTUs were detected. After removing *T*. *matsutake* reads, we normalized sequences to 570 reads (secondly smallest number) for further analysis. Because only 179 reads remained after removing *T*. *matsutake* reads, G1D sample was excluded the fungal community analysis. All Good's coverage estimates were 0.97–0.99, indicating that sequencing depth was appropriate for representing fungal diversity (S1 Table). The richness (Chao1) was significantly lower in the *Tm*-dominant soil than in the *Tm*-minor soil (*Corrected P* = 0.037), while both of diversity and evenness (Shannon) indices were not significantly different (Shannon: *Corrected P* = 0.579; Equitability: *Corrected P* = 0.779) (Fig 1A and S1 Table). The diversity and evenness showed significant differences for geographies (Shannon: *Corrected P* = 0.042; Equitability: *Corrected P* = 0. 042), while the richness did not (*Corrected P* = 0.063). No index showed a significant difference of interaction effects for soil type and geography (Chao1: *Corrected P* = 0.621; Shannon: *Corrected P* = 0.525; Equitability: *Corrected P* = 0. 525). Among fungal phyla, the fOTU richness of Basidiomycota was significantly lower in the *Tm*-dominant soil than the *Tm*-minor soil (*Corrected P* = 0.029) (Fig 2A). However, the fOTU richness in the other phyla did not have significant differences (Fig 2A).

#### Bacterial diversity

A total of 40,558 reads and 1,639 bOTUs were detected. The number of sequences was normalized to 1300 reads for further analysis. All samples had Good's coverage that indicated sufficient sequencing depth for characterizing bacterial diversity (0.94–0.99) (S2 Table). All indices of Chao1, Shannon diversity, Equitability, and phylogenetic diversity (PD) were significantly lower in the *Tm*-dominant soil than *Tm*-minor soil samples (Chao1: *Corrected P* = 0.026; Shannon: *Corrected P* = 0.005; Equitability: *Corrected P* = 0.014; PD: *Corrected P* = 0.005) (Fig 1B and S2 Table). Similarly, all indices were significantly different between geographies (Shannon: *Corrected P* = 0.017; Equitability: *Corrected P* = 0.033; PD: *Corrected P* = 0.019), except for Chao1 richness (*Corrected P* = 0.086). There were no significant interaction effects for soil type and geography. In comparing bOTU richness at the phyla level between soil types, Firmicutes was significantly higher in the *Tm*-dominant soil (*Corrected P* = 0.048), while Acidobacteria, Proteobacteria, and Verrucomicrobia were significantly higher in the *Tm*-minor soil (*Corrected P*: 0.048, 0.048, and 0.048, respectively) (Fig 2B).

### Microbial community structure associated with *T*. *matsutake*

#### Fungal community

**Fig 3:**
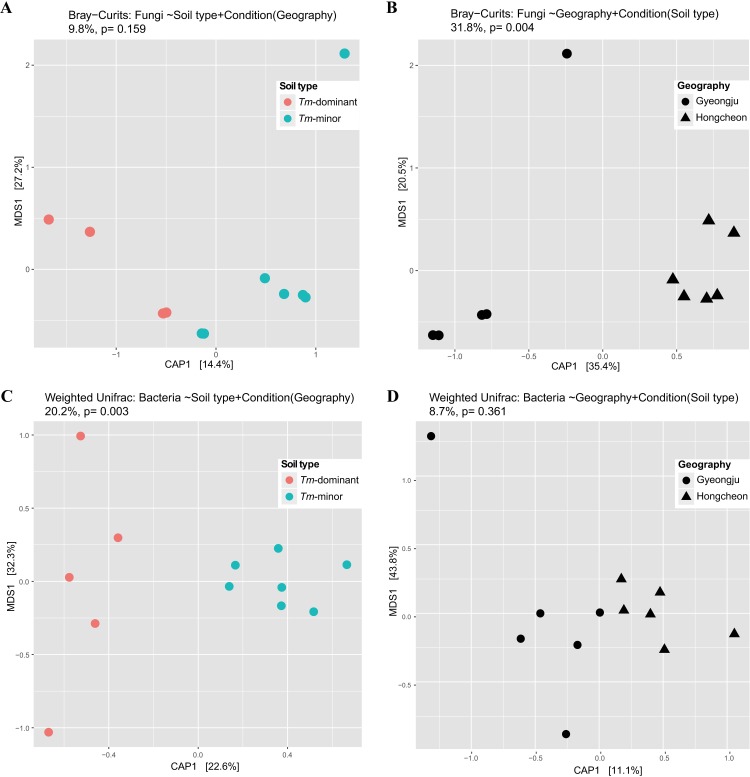
Constrained Analysis of Principal coordinates (CAP) plots for microbial communities in the *Tm*-dominant and *Tm*-minor soil. CAP model for (A) fungal and (C) bacterial communities constrained by soil type and conditioned by geographic location. CAP models for (B) fungal and (D) bacterial communities constrained by geographic location and conditioned by soil type. CAP analyses were conducted on Bray-Curtis distances for fungal communities and weighted Unifrac distances for bacterial communities. Significance of CAP models was evaluated using ANOVA with 999 permutations.

In the ordination analysis, points clustered based on soil type and geography. CAP analyses found the combination of soil type and geography to have a significant effect (*P* = 0.004) and explained 42.0% of the clustering pattern (S1A Fig). Geography alone had a significant effect on clustering (*P* = 0.004, 31.8% explanatory power), while soil type alone did not (*P* = 0.159, 9.8% explanatory power) (Fig 3A and 3B). PERMANOVA results were in agreement, indicating that the samples were differed significantly according to geography (*P* = 0.003), while soil type did not (*P* = 0.271).

The results from the discovery odds ratio test indicated that some fungal genera had significantly different abundance patterns between soil types across geographies (Table 1 and S2A Fig). Among the major genera, *Umbelopsis* was significantly more abundant in the *Tm*-dominant soil than the *Tm*-minor soil. In contrast, *Mortierella* was significantly more abundant in the *Tm*-minor soil.

**Table 1:** Microbial genera list of significantly enriched in a discovery odds ratio test. P-values were corrected to false discovery rates using the Benjamini and Hochberg method.

	Kingdom	Phylum	Genus	log_2_OR^*^	Corrected *P*
***Tm*-dominant**	Fungi	Zygomycota	*Umbelopsis*	1.24	3.49e-05
	Bacteria	Actinobacteria	*Mycobacterium*	1.19	7.13e-14
		Firmicutes	*Bacillus*	2.68	1.35e-15
		Firmicutes	*Paenibacillus*	5.13	3.90e-46
		Proteobacteria	*Burkholderia*	2.40	4.96e-34
***Tm*-minor**	Fungi	Zygomycota	*Mortierella*	-1.27	2.64e-03
	Bacteria	Acidobacteria	*Koribacter*	-1.50	3.43e-17
		Acidobacteria	*Solibacter*	-2.05	2.18e-23

*OR: Odds ratio

#### Bacterial community

Bacterial communities also clustered according to soil type and geography. In CAP analyses, the combination of soil type and geography showed a significant effect (*P* = 0.002) and explained 30.9% of the clustering pattern (S1B Fig). When analyzed separately for each factor, only soil type had a significant effect on clustering (*P* = 0.003, 20.2% explanatory power), while geography was not significant (*P* = 0.361) (Fig 3C and 3D). PERMANOVA tests supported this result, showing that bacterial communities were significantly different by soil type (*P* = 0.002), but was not by geography (*P* = 0.154).

The results of a discovery odds ratio test showed that some bacterial genera were differentially abundant between soil types (Table 1 and S2B Fig). *Bacillus*, *Burkholderia*, *Mycobacterium*, and *Paenibacillus* were significantly more abundant in the *Tm*-dominant soil, while *Koribacter* and *Solibacter* were significantly more abundant in the *Tm*-minor soil.

### Predicted functional profiles of bacterial communities associated with *T*. *matsutake*

We predicted the functional profiles of bacterial communities with PICRUSt. Considering that we were studying novel environmental communities, the statistical relevance of prediction was relatively well supported (NSTI = 0.03–0.17). Functional profiles were significantly different between the soil types. Six level-4 KEGG modules were statistically more abundant in the *Tm*-dominant soil, while none of significantly abundant modules were detected in the *Tm*-minor soil (Table 2). For these six modules, three modules were a Type III secretion system (T3SS) and effectors (M00332, M00660, and M00542), two were amino acid uptake (M00225 and M00226), and one involved in carbohydrate metabolism (M00519) (Table 2).

**Table 2:** List of KEGG modules that were significantly abundant in the *Tm*-dominant soil. Fold change is for *Tm*-dominant soil samples relative to *Tm*-minor soil samples. Counts per million (CPM) were calculated with normalized abundances using edgeR’s TMM method. P-values were corrected to false discovery rates using the Benjamini and Hochberg method.

KEGG module ID	Putative function	Fold change	log_2_CPM	Corrected *P*
M00519 YesM-YesN two-component regulatory system	Carbohydrate metabolism	5.47	7.13	5.92e-05
M00332 Type III secretion system	Type III secretion system	4.80	8.93	4.71e-05
M00660 Xanthomonas spp. pathogenicity signature, T3SS and effectors		4.71	8.66	4.71e-05
M00542 EHEC/EPEC pathogenicity signature, T3SS and effectors		4.64	8.69	4.71e-05
M00225 Lysine/arginine/ornithine transport system	Amino acid uptake	4.49	8.09	1.89e-04
M00226 Histidine transport system		4.19	7.71	7.39e-05

## Discussion

Hyphal dominant environments (often named mycosphere, mycorrhizosphere, ectomycorrhizal mat, and fairy ring) influence both abiotic and biotic factors in the soil ecosystems [29, 50–54]. *Tricholoma matsutake* forms its hyphal dominant environment around the fairy ring. Although the hyphal dominant environment can influence microbial communities [55], microbial interaction and functional activity in the *Tm*-dominant soil remain poorly characterized. In the work reported here, we compared soil communities between soil types (*Tm*-dominant and *Tm*-minor soil) across geographic locations. Our results showed an influence of *T*. *matsutake* dominance on the microbial diversity and community structure of other soil microbes as well as predicted bacterial functional profiles.

### Microbial diversity in the *Tm*-dominant soil

Consistent with most of previous studies (e.g., Ohara and Hamada [10]), we found that both fungal and bacterial diversity were significantly lower in the *Tm*-dominant soil than in the *Tm*-minor soil. However, this is in contrast to Kim et al. [15], who used a similar NGS approach as us, but found equal levels of bacterial diversity between the fairy ring and non-fairy ring zones in Korea. While the differences between our results and those of Kim et al. [15] could reflect a difference between geographic location, we consider this unlikely, given that previous studies in Japan [10, 13] also found lower bacterial diversity in the fairy ring. A more likely explanation is that the results of Kim et al. [15] reflect different sampling methods. Kim et al. [15] combined multiple samples from each of the three zones (inside, beneath, and outside a fairy ring). In our experience, morphological soil classification may be inconsistent with the dominance of *T*. *matsutake*; Kim et al. [15] may have combined samples from different soil types, which would have obscured the pattern of lower bacterial richness in the fairy ring.

As noted above, the low fungal richness in the *Tm*-dominant soil we found is consistent with previous studies [11, 14]. Because we excluded *T*. *matsutake* when calculating diversity indices, the dominance effect of low fungal richness is not simply a result of *T*. *matsutake* abundance. Although the Shannon’s diversity and evenness of fungal community were not significantly different between the *Tm*-dominant and *Tm*-minor soil, the dominance effect of *T*. *matsutake* likely reduced fungal richness. At the phylum level, Basidiomycota showed significant differences in fOTU richness between the soil types (Fig 2A). Considering that a large proportion of ectomycorrhizal fungi belong to Basidiomycota [56], the reduction of Basidiomycota richness may reflect exclusion by *T*. *matsutake*. Similar to other ectomycorrhizal fungi [57, 58], *T*. *matsutake* can secrete antifungal compounds [59] to exclude other fungal species, promoting its own fitness by reducing competitors.

Our results are consistent with previous studies showing that bacterial diversity is reduced in *Tm*-dominant soil across geographical locations [10, 13]. This result is also consistent with mycosphere studies under the fruiting body of another ectomycorrhizal fungus (*Laccaria proxima*) [54, 60]. The major difference we found between the *Tm*-dominant soil and *Tm*-minor soil was a reduction of the bOTU richness of the phyla Acidobacteria and Proteobacteria (Fig 2B). These phyla are abundant in soil environments [61, 62], but reduced richness in the *Tm*-dominant soil could be the result of a negative effect of *T*. *matsutake*, such as competition for resources or secretion of antibiotics to exclude bacteria [1]. In contrast, the bOTU richness of Firmicutes was higher in *Tm*-dominant soil. Firmicutes contains the genera *Bacillus*, *Cohnella*, and *Paenibacillus*, which are commonly found in mycorrhiza-associated environments [63–65]. Therefore, higher richness of Firmicutes may suggest a conserved trait associated with fungi-bacteria interaction or resistance to antibiotics. The interrelationship between these genera and *T*. *matsutake* is discussed below.

As shown in the *Tm*-dominant soil, low microbial diversity may be a general feature in hyphal dominant environments. In the mycosphere of *Laccaria*, bacterial diversity was significantly reduced compared to the bulk soil [66]. On the other hand, fungal diversity was also relatively low in the mycorrhizospheres of *Amanita* and *Laccaria* [67]. This trend is not limited to ectomycorrhizal fungi. In the mycosphere soil of several saprophytic mushrooms (Lachnocladiaceae, Lepiotaceae, and Marasmiaceae), pyrosequencing detected reduced microbial diversity [68]. However, some fungi showed higher or unchanged diversity in the mycosphere or mycorrhizosphere soil [67]. Therefore, reduction of microbial diversity is likely a species-specific feature among fungi such as *T*. *matsutake*. This alteration of microbial diversity between soil types suggests that the hyphae of *T*. *matsutake* can shape distinctive environments different from the adjacent soil. In addition, this environment can be harsh (as in those of Basidiomycota, Acidobacteria, Proteobactera, and Verrucomicrobia) or favorable (as in those of Firmicutes), depending on the taxa, which suggest that this is a conserved trait.

### Distinct microbial community structure

If the microbial community associated with the *Tm*-dominant soil is distinct and shared across geographic locations, the dominance effect of *T*. *matsutake* may cause spatially distinct microbial communities to become more similar. CAP and PERMANNOVA analyses showed different trends in fungal and bacterial communities. Bacterial communities were significantly different between the *Tm*-dominant soil and *Tm*-minor soil, and shared community structure within soil types, while fungal community was not (Fig 3A and 3C). This suggests that dominance of *T*. *matsutake* may have stronger influence on bacterial communities than on fungal communities. If *T*. *matsutake* has a negative effect on microbial diversity, genera that were enriched in the *Tm*-dominant soil may have a specific relationship with *T*. *matsutake*.

Although the fungal community structure was not different between soil types, *Umbelopsis* was significantly more abundant in *Tm*-dominant soil samples across geographic locations (Table 1 and S2A Fig). *Umbelopsis* was frequently detected from the fruiting body and the fairy ring of *T*. *matsutake* [13, 69, 70]. Because *Umbelopsis* species formerly belonged to *Mortierella*, *Umbelopsis* isolated from the fairy ring was identified as *Mortierella* [71]. However, our result showed that *Mortierella* and *Umbelopsis* have opposite distribution trends associated with *T*. *matsutake*. *Umbelopsis* may have positive interactions with *T*. *matsutake*. In addition, *Umbelopsis* is likely a helper fungus that promotes growth of *T*. *matsutake*; a previous study showed that metabolites from “*Mortierella*” *nana* (= *Umbelopsis nana*) enhanced hyphal growth of *T*. *matsutake* [72].

Bacterial communities also showed distinct community structures in the different soil types (Fig 3C). *Bacillus*, *Burkholderia*, *Mycobacterium*, and *Paenibacillus* were statistically more abundant in the *Tm*-dominant soil than the *Tm*-minor soil (Table 1 and S2B Fig). Most of these genera were previously detected in the fairy ring of *T*. *matsutake* [13, 15], and are commonly found in the rhizosphere or mycorrhizosphere [63–65, 73]. In addition, these taxa may be similar on a functional level. For example, *Bacillus*, *Burkholderia*, and *Paenibacillus* are known to be mycorrhiza helper bacteria (MHB) that promote the growth and colonization of mycorrhizae [74–76]. Co-occurrence suggests that *T*. *matsutake* may recruit mutualistic microbes as part of its dominance effect. On the other hand, these bacteria actively orientate to *Tm*-dominant soil for exploiting nutrient from *T*. *matsutake*. Diverse nutrients are exuded from hyphae (e.g. carbon hydrates from plant) [54], and hyphae can be a nutrient source itself for the mycophagous bacteria [77]. Therefore, bacteria abundant in the *Tm*-dominant soil are either recruited by *T*. *matsutake*, gather by themselves, or a combination of both.

Distinct microbial communities and enrichment of specific microbes in the mycorrhizosphere and mycosphere have been reported [66, 78]. Among the bacteria enriched in the hyphal dominant environment, *Burkholderia*, *Pseudomonas*, and *Variovorax* associated with various fungi [54, 66, 79–81], thus, these bacteria can be classified as fungiphilic bacteria [66]. Enrichment of fungiphiles in the hyphal dominant environment is thought to be connected with specific traits. For example, fungiphiles can use surface of hyphae to move on like a highway [82, 83], and have T3SS mediating interactions with fungi [60]. Moreover, fungiphiles can use the material excreted from the hyphae (*e*.*g*. carbohydrates [maltose and trehalose] and amino acids [alaninamine and ornithine]) as an energy source [66]. Fungal communities are also different in the hyphal dominant environment [80, 84]. In addition, Sabella et al. [85] showed the potential of mycorrhizal helper fungus (*Arthrinium phaeospermum* for *Tuber*) like MHB. Therefore, mutualistic fungi may co-exist in the hyphal dominant environment, and our results highlighted *Umbelopsis* as a likely mutualistic fungus.

### Functional association of distinct bacterial community

We analyzed whether the bacterial community of the *Tm*-dominant soil produces a distinct functional profile in the soil. If true, this would link *T*. *matsutake* to specific functions of the soil microbiota. The low NSTI value (0.03–0.17) from our PICRUSt analysis indicated a reasonable accuracy of prediction. Our results indicated that *Tm*-dominant soil and *Tm*-minor soil were functionally distinct. The six functional modules found to be statistically higher in the *Tm*-dominant soil than in the *Tm*-minor soil can be organized into two groups based on their putative functions: nutrient uptake (amino acid and carbohydrate) and type III secretion systems (T3SS). These functions can be associated with microbial interaction and exploitation by increasing bacterial survival and mediating interactions between bacteria and *T*. *matsutake*. For the first effect, improved bacterial survival can be facilitated by uptake of amino acids (arginine, histidine, lysine, and ornithine) and carbohydrates (Table 2). As mentioned above, an increase of nutrient metabolism associated modules suggests that these bacteria favor *Tm*-dominant soil because bacteria in the *Tm*-dominant soil may more easily acquire nutrients.

The second effect, mediation of *T*. *matsutake*-bacteria interactions, could be enhanced by T3SS. While T3SS is a well known system for mediating bacterial interactions with various eukaryotes [86], T3SS is also crucial for fungi-bacteria interactions [87]. Studies of bacteria associated with mycorrhizal fungi found increased numbers of T3SS genes in both the mycorrhizosphere and mycosphere [60, 88]. Additionally, T3SS of *Pseudomonas fluorescens* has an MHB effect, enhancing growth of *Laccaria bicolor* [89].

By characterizing functional profiles of bacteria in the *Tm*-dominant soil, we identified several functional modules that were potentially associated with microbial interactions with *T*. *matsutake*. Increased functions associated with nutrient uptake suggest that microbial interaction in the *Tm*-dominant soil may improve bacterial fitness. However, the benefit for *T*. *matsutake* remains unknown. Although there are limits to the interpretation of functional profile predictions, we have identified functions with potential positive effects. Future studies can target these functions to clarify the dynamics between organisms in the *Tm*-dominant soil.

In contrast to our results, the studies on the functional traits in the hyphal dominant environment showed an increase in traits not only for bacterial fitness, but also for the hyphal host's fitness. This may be explained by previous studies. For examples, *Pseudomonas fluorescens* promotes hyphal growth [90] and ectomycorrhizal formation of *Laccaria bicolor* [91], and T3SS (enriched modules in the *Tm*-dominant soil) is essential component to promoting effect [89]. Moreover, it is possible to help fungi indirectly by improving fungi-plant interaction. For example, transferring nitrogen and phosphate is important in ectomycorrhiza-plant relationships. Bacteria that can carry out mineral weathering can enhance ectomycorrhizal capability [92, 93]. Although culture-dependent and gene targeted studies showed distinct functional traits in the hyphal dominant environment, there are limited studies of functional traits using metagenomic or metatranscriptomic approaches because of cost and complexity of soil environment [20, 21]. As we have shown, predicted functional profiles using PICRUSt can be an intermediate method using targeted amplicon datasets for glancing overall functional profiles in metagenomic samples.

## Conclusions

We compared *Tm*-dominant soil and *Tm*-minor soil samples across geographic locations in order to investigate possible dominance effects of *T*. *matsutake*. The similarity of microbial communities and bacterial functional profiles in the *Tm*-dominant soil suggests that this fungus may have a dominance effect on soil microorganisms. We identified several dominant fungal (*Umbelopsis*) and bacteria genera (*Bacillus*, *Burkholderia*, *Mycobacterium*, and *Paenibacillus*) in the *Tm*-dominant soil that may exploit or help *T*. *matsutake*. Regarding functional analyses, we identified functional modules in the *Tm*-dominant soil that are associated with nutrient metabolism for bacteria and bacterial-fungal interactions. The *Tm*-dominant soil is a complex environment, and our work implies multiple symbiotic relationships between microorganisms—*Tricholoma matsutake* may shape bacterial/fungal diversity, while microorganisms associated with the *Tm*-dominant soil may enhance *T*. *matsutake* growth. As co-occurrence of microorganisms does not necessarily mean interaction, additional study of microbial taxa and functional roles is needed to clarify microbial interactions and functions caused by the dominance effect of *T*. *matsutake*. Nevertheless, we believe our study enhances the understanding of *T*. *matsutake* in terms of microbial interactions, and the dominance effect of *T*. *matsutake* could provide knowledge of fungal effect on the microbial community in soil ecosystems.

## Supporting Information

S1 Fig Constrained Analysis of Principal coordinates (CAP) plots for microbial communities in the *Tm*-dominant and *Tm*-minor soil. CAP model for (A) fungal and (B) bacterial communities, constrained by soil type and geographic location. CAP analyses were conducted on Bray-Curtis distances for fungi and weighted Unifrac distances for bacteria. Significance of CAP models was evaluated using ANOVA with 999 permutations.
(PDF)

S2 Fig Differential distribution of major microbial genera between soil types (the *Tm*-dominant and *Tm*-minor soil). Only genera that were present above 1% are shown for (A) fungi and (B) bacteria. Significance of differential abundance between the *Tm*-dominant and *Tm*-minor soil was evaluated using a discovery odds ratio test. The genera that were significantly different between soil types (*Corrected P* < 0.05) are represented with asterisk. Genera are grouped based on phylum membership (Basi: Basidiomycoota, Zygo: Zygomycota, Acid: Acidobacteria, Acti: Actinobacteria, Chlo: Chloroflexi, Firm: Firmicutes, Prot: Proteobacteria, Verr: Verrucomicrobia).
(PDF)

S1 Table Information on the fungal communities for soil samples. Diversity indices and Good’s coverage were calculated after normalization (570 reads) without *T*. *matsutake* reads. Because the number of G1D sequences without *T*. *matsutake* reads was too small, G1D was excluded for diversity measurement.
(DOCX)

S2 Table Information on the bacterial communities for soil samples. Diversity indices and Good’s coverage were calculated after normalization (1300 reads).
(DOCX)
