# Supplementary figures and images for: Distinctive Feature of Microbial Communities and Bacterial Functional Profiles in Tricholoma matsutake Dominant Soil
Source: PLoS One. 2016 Dec 15;11(12):e0168573. doi: 10.1371/journal.pone.0168573 (PMC5158061; doi:10.1371/journal.pone.0168573)

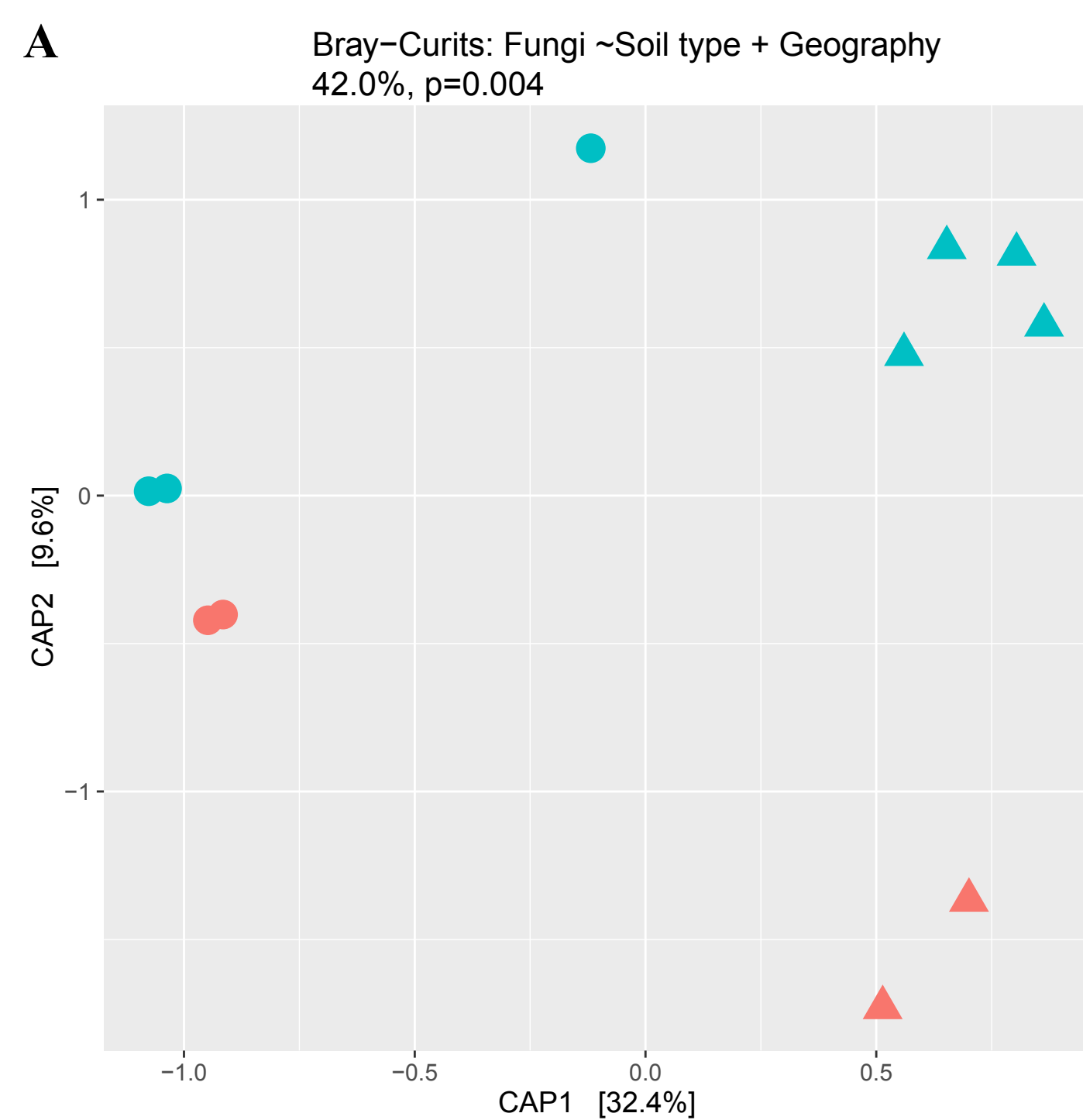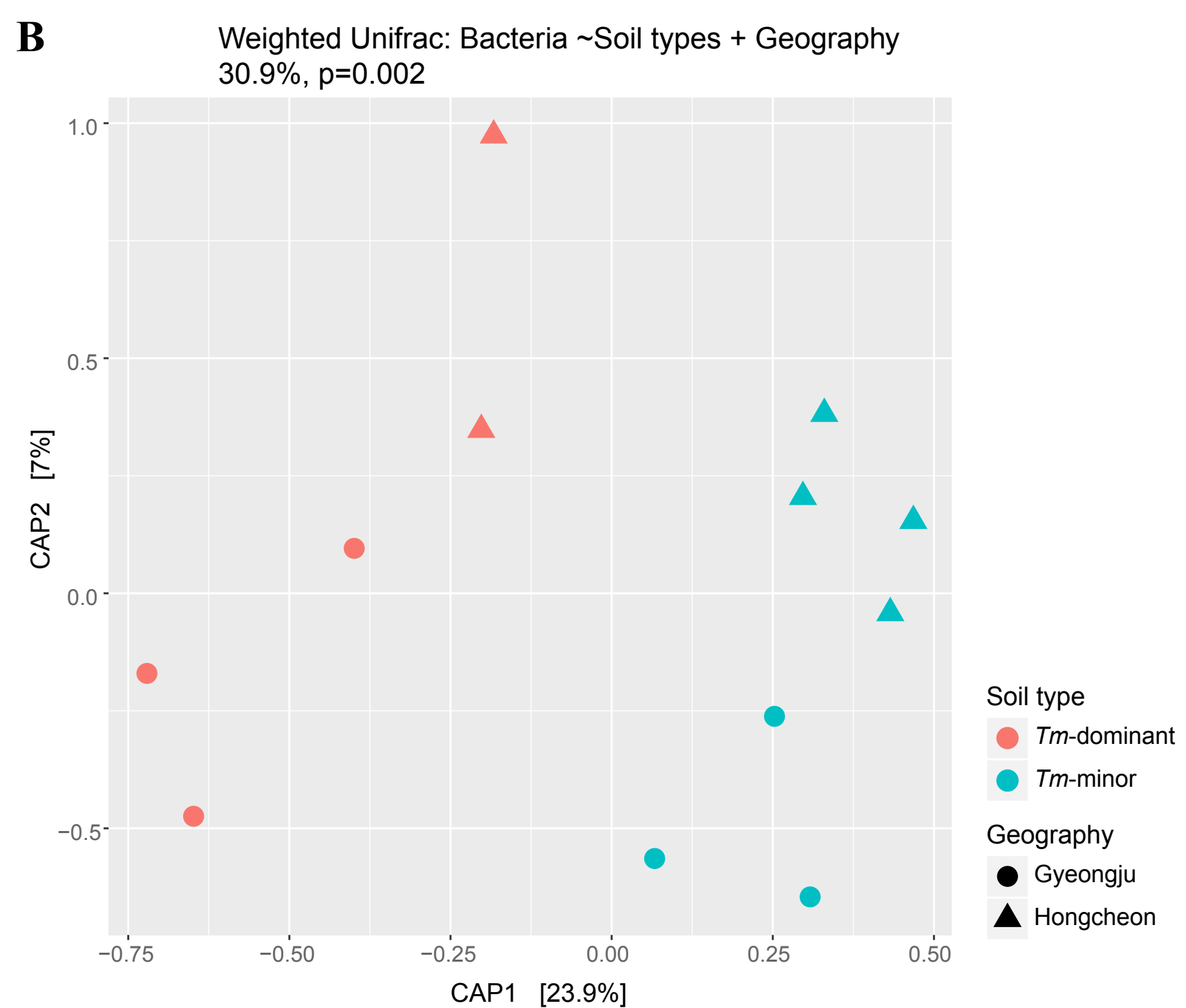

Supplement: S1 Fig — CAP model for (A) fungal and (B) bacterial communities, constrained by soil type and geographic location. CAP analyses were conducted on Bray-Curtis distances for fungi and weighted Unifrac distances for bacteria. Significance of CAP models was evaluated using ANOVA with 999 permutations. (PDF) [file pone.0168573.s001.pdf]

**A**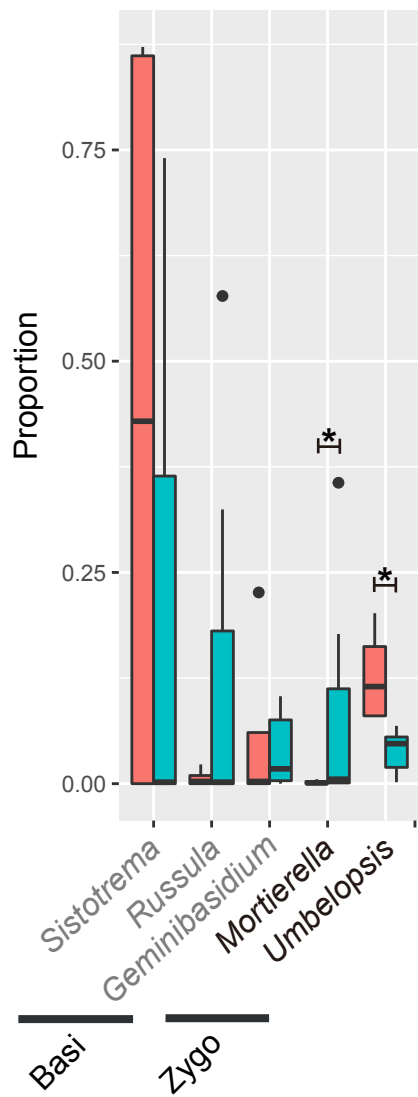**B**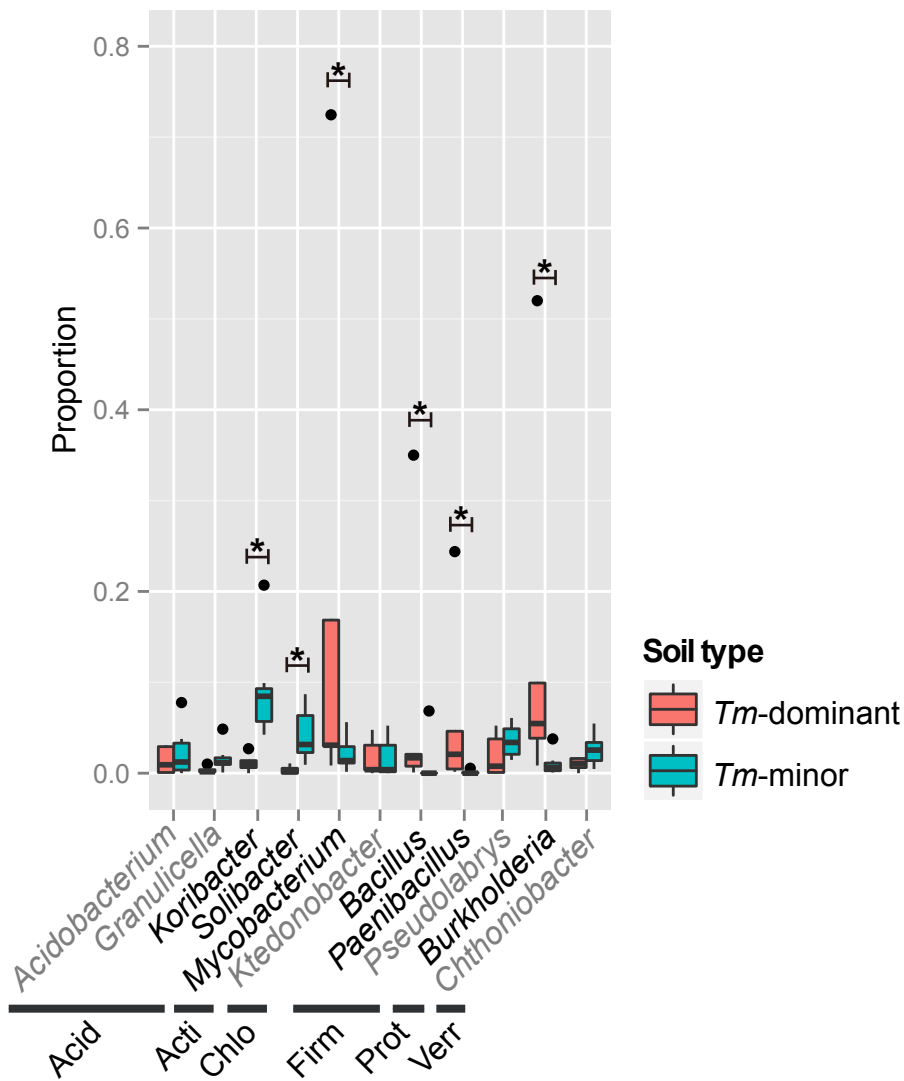

Supplement: S2 Fig — Only genera that were present above 1% are shown for (A) fungi and (B) bacteria. Significance of differential abundance between the Tm-dominant and Tm-minor soil was evaluated using a discovery odds ratio test. The genera that were significantly different between soil types (Corrected P < 0.05) are represented with asterisk. Genera are grouped based on phylum membership (Basi: Basidiomycoota, Zygo: Zygomycota, Acid: Acidobacteria, Acti: Actinobacteria, Chlo: Chloroflexi, Firm: Firmicutes, Prot: Proteobacteria, Verr: Verrucomicrobia). (PDF) [file pone.0168573.s002.pdf]
