# Supplementary material for: Distinctive Feature of Microbial Communities and Bacterial Functional Profiles in Tricholoma matsutake Dominant Soil
Source: PLoS One. 2016 Dec 15;11(12):e0168573. doi: 10.1371/journal.pone.0168573 (PMC5158061; doi:10.1371/journal.pone.0168573)
Supplement: S1 Table — Diversity indices and Good’s coverage were calculated after normalization (570 reads) without T. matsutake reads. Because the number of G1D sequences without T. matsutake reads was too small, G1D was excluded for diversity measurement. (DOCX) [file pone.0168573.s003.docx]

**S1 Table. Information on the fungal communities for soil samples. Diversity indices and Good’s coverage were calculated after normalization (570 reads) without *T. matsutake* reads. Because the number of G1D sequences without *T. matsutake* reads was too small, G1D was excluded for diversity measurement.**

| **Soil type** | **Sample ID** | **Location** | **SR. No.^*^** | ***Tm***^†^ **Proportion (%)** | **Diversity measure** | | | **Good's coverage** |
| --- | --- | --- | --- | --- | --- | --- | --- | --- |
|  |  |  |  |  | **Chao1** | **Shannon** | **Shannon’s equitability** |  |
| ***Tm*-dominant** | G1D | Gyeongju | 9284 | 98.1% | - | - | - | - |
|  | G2D | Gyeongju | 4448 | 87.2% | 14.00 | 0.81 | 0.23 | 0.99 |
|  | G3D | Gyeongju | 7240 | 84.8% | 9.50 | 0.88 | 0.28 | 0.99 |
|  | H2D | Hongcheon | 5956 | 88.7% | 25.00 | 2.70 | 0.63 | 0.98 |
|  | H5D | Hongcheon | 4500 | 83.6% | 34.14 | 2.72 | 0.56 | 0.98 |
|  |  |  |  |  |  |  |  |  |
| ***Tm*-minor** | G4m | Gyeongju | 11997 | 0.0% | 25.00 | 1.52 | 0.36 | 0.98 |
|  | G5m | Gyeongju | 2229 | 34.7% | 41.00 | 1.92 | 0.41 | 0.98 |
|  | G6m | Gyeongju | 9094 | 1.3% | 43.75 | 1.83 | 0.36 | 0.97 |
|  | H1m | Hongcheon | 4263 | 0.0% | 40.00 | 3.46 | 0.68 | 0.98 |
|  | H3m | Hongcheon | 7283 | 0.0% | 36.14 | 2.54 | 0.51 | 0.98 |
|  | H4m | Hongcheon | 4180 | 0.0% | 64.00 | 3.61 | 0.66 | 0.97 |
|  | H6m | Hongcheon | 5820 | 0.0% | 51.00 | 1.19 | 0.26 | 0.97 |

*^*^* SR. No: Sequence reads number
^†^*Tm*: *Tricholoma matsutake*
