# Supplementary material for: Distinctive Feature of Microbial Communities and Bacterial Functional Profiles in Tricholoma matsutake Dominant Soil
Source: PLoS One. 2016 Dec 15;11(12):e0168573. doi: 10.1371/journal.pone.0168573 (PMC5158061; doi:10.1371/journal.pone.0168573)
Supplement: S2 Table — Diversity indices and Good’s coverage were calculated after normalization (1300 reads). (DOCX) [file pone.0168573.s004.docx]

**S2 Table. Information on the bacterial communities for soil samples. Diversity indices and Good’s coverage were calculated after normalization (1300 reads).**

| **Soil type** | **Sample ID** | **Location** | **SR. No.^*^** |  | **Diversity measure** | | | | **Good's coverage** |
| --- | --- | --- | --- | --- | --- | --- | --- | --- | --- |
|  |  |  |  |  | **Chao1** | **Shannon** | **Shannon’s equitability** | **PD**^†^ |  |
| ***Tm*-dominant** | G1D | Gyeongju | 4550 |  | 122.00 | 2.96 | 0.45 | 10.73 | 0.98 |
|  | G2D | Gyeongju | 4397 |  | 96.67 | 3.82 | 0.62 | 7.89 | 0.99 |
|  | G3D | Gyeongju | 2501 |  | 193.07 | 5.16 | 0.74 | 13.47 | 0.97 |
|  | H2D | Hongcheon | 1355 |  | 207.04 | 6.24 | 0.85 | 13.08 | 0.96 |
|  | H5D | Hongcheon | 2252 |  | 321.67 | 6.14 | 0.81 | 16.58 | 0.96 |
|  |  |  |  |  |  |  |  |  |  |
| ***Tm*-minor** | G4m | Gyeongju | 4590 |  | 218.71 | 6.04 | 0.82 | 13.16 | 0.97 |
|  | G5m | Gyeongju | 5505 |  | 227.00 | 5.92 | 0.80 | 14.40 | 0.97 |
|  | G6m | Gyeongju | 4858 |  | 347.56 | 6.36 | 0.83 | 18.64 | 0.96 |
|  | H1m | Hongcheon | 2086 |  | 280.67 | 6.71 | 0.86 | 19.75 | 0.95 |
|  | H3m | Hongcheon | 2247 |  | 294.67 | 6.89 | 0.88 | 20.05 | 0.94 |
|  | H4m | Hongcheon | 1894 |  | 266.81 | 6.42 | 0.84 | 21.02 | 0.96 |
|  | H6m | Hongcheon | 4323 |  | 272.88 | 6.60 | 0.84 | 18.63 | 0.95 |

*^*^* SR. No: Sequence reads number
^†^PD: phylogenetic diversity index (Faith, 1992).
